# Tracing the Progression of Sepsis in Critically Ill Children: Clinical Decision Support for Detection of Hematologic Dysfunction

**DOI:** 10.1055/a-1950-9637

**Published:** 2022-10-26

**Authors:** Louisa Bode, Sven Schamer, Julia Böhnke, Oliver Johannes Bott, Michael Marschollek, Thomas Jack, Antje Wulff, ELISE Study Group

**Affiliations:** 1 Peter L. Reichertz Institute for Medical Informatics of TU Braunschweig and Hannover Medical School, Hannover, Germany; 2 Department of Pediatric Cardiology and Intensive Care Medicine, Hannover Medical School, Germany; 3 Institute of Epidemiology and Social Medicine, University of Muenster, Muenster, Germany; 4 University of Applied Sciences and Arts Hannover, Faculty III – Media, Information and Design, Hannover, Germany; 5 Big Data in Medicine, Department of Health Services Research, School of Medicine and Health Sciences, Carl von Ossietzky University Oldenburg, Oldenburg, Germany

**Keywords:** decision support systems, clinical, hematology, openEHR, organ failure, intensive care units, pediatric, diagnostic accuracy

## Abstract

**Background** One of the major challenges in pediatric intensive care is the detection of life-threatening health conditions under acute time constraints and performance pressure. This includes the assessment of pediatric organ dysfunction (OD) that demands extraordinary clinical expertise and the clinician's ability to derive a decision based on multiple information and data sources. Clinical decision support systems (CDSS) offer a solution to support medical staff in stressful routine work. Simultaneously, detection of OD by using computerized decision support approaches has been scarcely investigated, especially not in pediatrics.

**Objectives** The aim of the study is to enhance an existing, interoperable, and rule-based CDSS prototype for tracing the progression of sepsis in critically ill children by augmenting it with the capability to detect SIRS/sepsis-associated hematologic OD, and to determine its diagnostic accuracy.

**Methods** We reproduced an interoperable CDSS approach previously introduced by our working group: (1) a knowledge model was designed by following the commonKADS methodology, (2) routine care data was semantically standardized and harmonized using openEHR as clinical information standard, (3) rules were formulated and implemented in a business rule management system. Data from a prospective diagnostic study, including 168 patients, was used to estimate the diagnostic accuracy of the rule-based CDSS using the clinicians' diagnoses as reference.

**Results** We successfully enhanced an existing interoperable CDSS concept with the new task of detecting SIRS/sepsis-associated hematologic OD. We modeled openEHR templates, integrated and standardized routine data, developed a rule-based, interoperable model, and demonstrated its accuracy. The CDSS detected hematologic OD with a sensitivity of 0.821 (95% CI: 0.708–0.904) and a specificity of 0.970 (95% CI: 0.942–0.987).

**Conclusion** We could confirm our approach for designing an interoperable CDSS as reproducible and transferable to other critical diseases. Our findings are of direct practical relevance, as they present one of the first interoperable CDSS modules that detect pediatric SIRS/sepsis-associated hematologic OD.

## Background and Significance

Diagnosing organ dysfunction (OD) along the clinical pathway of critically ill children suffering from systemic inflammatory response syndrome (SIRS) or sepsis is a knowledge-intensive and fault-prone task that clinicians have to solve under profound urgency, due to its life-threatening character. 1 SIRS can progress to sepsis if there is an infection. As the name already suggests, SIRS represents a systemic response of the entire body often resulting in further suffering of affected organ systems. OD and failures occur, leading to a decreased chance of full recovery. By early initiation of antibiotic or symptomatic treatment continuously along this pathway, fatal outcomes might be preventable and mortality might be reduced. Thus, besides detecting SIRS as the starting point, also the early detection of associated OD along the path is important since it allows timely assessment of the progression state of infection in the patient. 2 Additionally, by this separate analysis for the presence of any OD, an early differentiation between SIRS and severe SIRS is possible. In 2005, field experts and researchers at the International Pediatric Sepsis Consensus Conference (IPSCC) 3 agreed on a set of diagnostic criteria for sepsis and associated ODs, including the multiple organ dysfunction syndrome (MODS). Concerning pediatric OD, the criteria comprise a list of laboratory parameters and thresholds for each organ system (respiratory, cardiovascular, renal, hepatic, hematologic, and neurological) potentially affected. 3 For the hematologic system of children, two physiological parameters are of great relevance: the platelet count and the international normalized ratio (INR). 3 According to Goldstein et al, 3 pediatric hematologic OD is defined as:

- “Platelet count <80,000/mm ^3^ or a decline of 50% in platelet count from the highest value recorded over the past 3 days (for chronic hematology/oncology patients).
- Or, an international normalized ratio >2.

Delayed diagnosis can increase mortality, either by increasing the severity of OD or by increasing the number of failing organs. 4 Depending on the admission diagnosis, mortality rates range from approximately 14.3 to 24.7%. 5

Concomitant with a fast-growing amount of complex and heterogeneous data, there is an increasing interest in clinical decision support (CDS). 6 However, for a CDSS to enhance its usefulness at the point of care, it must be well integrated into the clinical workflow. In related work, best practices and lessons learned describe key aspects of a successful implementation, focusing on user-friendly designs and evidence-based-driven algorithms. 7 8 9 10 To decide on the presence or absence of a physiological abnormality, physicians have to interpret different physiological parameters from various information sources, such as the laboratory information system, electronic health record (EHR), or a ward-specific patient data management system (PDMS). These data need to be analyzed for each patient individually as normal ranges vary between age groups. 11 Altogether, various aspects need to be considered in a short period, requiring substantial practical experience. Taking these unique conditions into account, clinical decision support systems (CDSS) could provide added value in reducing the workload of staff and enhancing a patient's condition by providing more accurate diagnoses. 12 13 14 To make sure that the decisions of the CDSS are reliable, a precise knowledge base is essential to cover all clinical and patient-related details that are crucial for making decisions fitting best to the patient's health and well-being. 15 Furthermore, to allow broad applicability and sharing of CDSS, semantic interoperability standards, interfaces, and FAIR principles 16 should be considered. This includes the reuse of existing data in a standardized and semantically enriched form rather than building yet another stand-alone system (specifically designed for an institution), implementing a new vendor locked-in PDMS, or relying on specific non-standardized data representations from a primary source system. To avoid this, various semantic interoperability or messaging standards are available, such as openEHR, 17 HL7 CDA/CCR, 18 or HL7 FHIR. 19

In our previous work, we successfully presented and evaluated a holistic approach for designing an interoperable, knowledge-based CDSS for early detection of SIRS in pediatric intensive care units (PICU). 20 In this work, we aim at reproducing and enhancing this interoperable and rule-based CDSS approach for the detection of pediatric, SIRS/sepsis-associated, hematologic OD, thus also exploring the transferability of our design approach.

## Objectives

Our long-term goal is the development of a CDSS able to assess patients with respect to sepsis. Therefore, we need to be able to identify pathological states that are clinically relevant for the clinical pathway of the patient along SIRS/sepsis progression, starting with initial SIRS detection 20 21 and going on with abnormal events related to SIRS/sepsis such as associated OD. In this paper, we follow our research path by focusing on the computerized detection of SIRS/sepsis-associated hematologic OD.

1. To enhance an existing, interoperable, and rule-based CDSS prototype for tracing the progression in critically ill children by augmenting it with the capability to detect SIRS/sepsis-associated hematologic dysfunction.
2. To determine diagnostic accuracy (i.e., sensitivity and specificity) of the CDSS for detecting pediatric hematologic dysfunction by analyzing real-world data as a proof-of-concept (PoC) study.

## Methods

### Interoperability in CDSS

We have reproduced an interoperable CDSS approach previously introduced by our working group for computerized detection of SIRS in PICU. A key characteristic of this concept is the use of openEHR as a clinical information standard. 20 OpenEHR specifies an open platform architecture for EHR systems and outmatches traditional approaches by ensuring semantic interoperability for collecting and exchanging clinical data. 22 23 This is assured as long as the exchanging institutions use the same standardized clinical information models, the so-called archetypes. Archetypes are used to define the clinical information to be represented (e.g., a laboratory test result) in a uniform, machine-readable as well as machine-actionable, and standardized form. 24 Thus, they are rich and computable metadata models of clinical information. Archetypes rely on the reuse of standardized and internationally agreed-upon data items but simultaneously encapsulate this technical content from the domain-specific aspects. 25 For example, for representing a laboratory test result, a standardized model, agreed upon internationally by various experts from different domains, can be used to capture and/or integrate routine laboratory test data. 26 Nesting and restricting different archetypes, the outcome is a template that usually refers to an entire document or form (e.g., laboratory report) that requires various archetypes to meet the local needs of an institution. 22 Terminology bindings (e.g., SNOMED-CT 27 or LOINC 28 ) are possible on both archetype and template levels. 29 Throughout the modeling process and subsequently, archetypes and templates can be uploaded, managed, reviewed, and accessed via a web-based data repository, the Clinical Knowledge Manager (CKM). 30 Cross-institutional data transfer does not depend on local infrastructures, applications, and vendors, but rather on a common reference model (RM) which does not rely on elusive clinical knowledge. 22 Instead, the RM represents the basis upon which software applications can be built. Thus, decision logic in software applications is separated from clinical data, ensuring vendor independence and interoperability on the application level. 23 For the use case of hematologic OD, appropriate templates, consisting of international agreed-upon archetypes available in the CKM, need to be modeled to map the diagnostic criteria.

### Step-wise Implementation Strategy

**Fig. 1:**
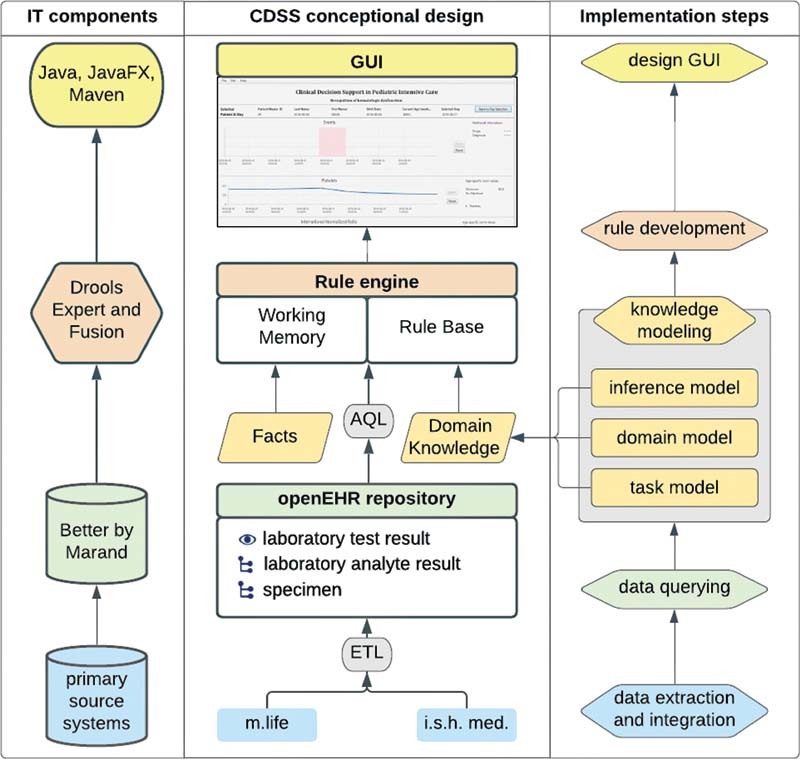
Bottom-up implementation plan to enrich the existing CDSS. CDSS, clinical decision support systems.

Fig. 1 shows an overview of the existing CDSS architecture and the necessary modeling and implementation steps adapted for our new use case. For more information on the existing CDSS architecture, we refer to Wulff et al. 20

#### Step 1: Data Extraction and Integration

Data used in the CDSS is routinely captured by the PDMS *m.life* a by medisite (version 11.2.4) of the PICU at Hannover Medical School (HMH). After the extraction of routine data from primary source systems, data were transformed and loaded into an openEHR data repository by using standardized data models. Currently, our data extraction and integration processes are performed only on demand. However, it is possible to adjust those processes to be performed every time a new value comes in. The openEHR data repository used was based on the Better platform by Marand. 31

#### Step 2: Data Querying

For retrieving data stored in archetypes, the model-based Archetype Query Language (AQL) was used. 32 AQL-constructed queries retrieve data from the openEHR data repository via a REST API and were – in contrast to SQL or similar query languages – independent of underlying primary source database structures and system vendors. This means that they work on any openEHR platform that implements the abovementioned, internationally agreed-upon archetypes.

#### Step 3: Knowledge Modeling

We adopted our previously designed approach for knowledge acquisition and representation that comprises the use of commonKADS as a well-documented and comprehensible methodology for acquiring, modeling, and processing knowledge. 33 In addition to literature research, we performed structured interviews to exchange information with two experienced intensive care pediatricians. The clinical question and methodology have been raised directly from the clinical context and worked out together with computer specialists. Knowledge transfer was the focus at the beginning. For this purpose, the pediatricians received a questionnaire tailored to the clinical picture of sepsis-associated hematological dysfunction. Based on these knowledge assets provided in the answers and additional expert discussions during rule development, the human-readable rules were jointly designed and translated into machine-readable rules by computer scientists (during step 4, see below). During the evaluation, pediatricians reviewed false positive and false negative alarms so that either the gold standard or the rule base could be optimized. We used commonKADS as a model-driven approach to conceptualize three knowledge models for each knowledge category including domain, inference, and task knowledge. 33 When knowledge was revealed at a later stage in the knowledge engineering process (e.g., during evaluation), the knowledge base was adapted iteratively. 15

#### Step 4: Rule Development

For transferring expert knowledge into computable problem-solving steps, we implemented rules by using the Business Rules Management System Drools by JBoss (Red Hat), 34 which provides techniques to embed reasoning structures.

#### Step 5: Graphical User Interface Design

For the visualization of vital signs series, laboratory value courses, and warnings, a graphical user interface based on JavaFX – a framework for developing applications built on Java 35 – was designed. We concentrated on making decisions explainable and visually easy to catch. The graphical user interface visualizes also parameters that are not directly relevant to hematologic dysfunction but all together are required to provide information about the clinical progression of SIRS/sepsis.

### Clinical Evaluation and Statistical Analysis

We used data from the prospective, monocentric, double-blinded, diagnostic CADDIE2 study to estimate diagnostic accuracy ( *sensitivity and specificity* ) of the rule-based CDSS ( *index test* ). Simultaneously, two experienced pediatricians defined the reference standard by blinded, retrospective digital chart review, and analysis based on the IPSCC criteria.

The CADDIE2 study included 168 consecutively sampled patients (0–18 years) from the interdisciplinary PICU of the MHH, who stayed for at least 12 hours with valid informed consent, between August 2018 to September 2019. These 168 patients reflected the intended sample size for the evaluation of the diagnostic accuracy of SIRS detection. For details on the study population and recruited patients, we refer to Wulff et al. 21

We divided the PICU stay of a patient into blocks that were then labeled as “true positive,” “false negative,” “true negative,” or “false positive” (for details see Supplementary Material S1 , available in the online version). The blocks were generated dependent on a change in the diagnostic status (OD present vs. no OD) of either the CDSS, the reference standard, or both simultaneously. Missing values or indeterminate results were not possible. However, the labeling was slightly modified by using a ± 4-hour window around the onset and end of the reference standard episode (exact timestamps defined by clinicians) as well as merging diagnoses with less than 24 hours between episodes. The period from the onset to the end of an episode is referred to as the diagnostic episode, i.e., the patient is diseased (for details see Supplementary Material S1 , available in the online version). Based on the labels, sensitivity and specificity with their Wald 95% confidence intervals (CI) were estimated using the approach by Brunner and Zapf, 36 Lange, 37 and Rooney, 38 which accounts for the longitudinal data format (several blocks per patient). The analysis was performed in R version 4.1.2 (November 01, 2021). A subgroup or sensitivity analysis was not conducted.

**Table 1:** Cross-tabulation of time blocks among 168 patients

	Clinician's diagnosis		
CDSS	Hematologic dysfunction Positive	Hematologic dysfunction Negative	Total
Alarm	55	8	63
No alarm	12	262	274
Total	67	270	337

Abbreviation: CDSS, clinical decision support systems.

## Results

### Conceptualized Knowledge Model for Hematologic Organ Dysfunction

**Fig. 2:**
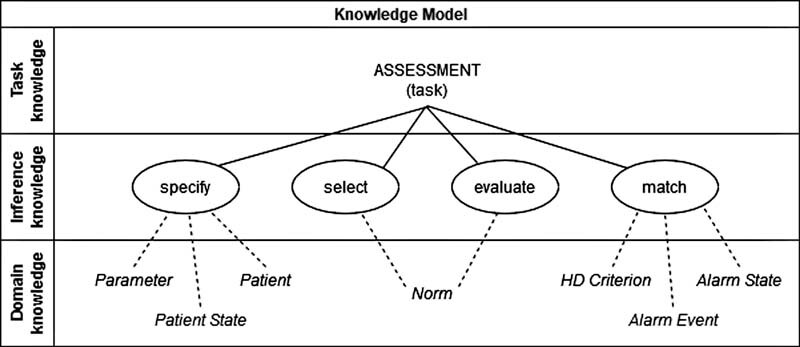
Simplified knowledge model and its dependencies of all three commonKADS knowledge categories.

The preparation of a problem- and application-specific knowledge base is of great importance. Therefore, we conceptualized a knowledge model ( Fig. 2 ) composed of a task, domain, and inference model. The inference model is required to perform problem-solving steps under the use of static expert knowledge (domain model) to complete the task of recognizing hematologic OD (task model). First, the normal range for each laboratory parameter must be *specified* (inference: specify). Depending on the underlying parameter, the correct normal range needs to be *selected* (inference: select), thereby in the next step, the respective laboratory result can be *evaluated* by comparing it to the normal range (inference: evaluate). Afterward, it can be inferred whether the value is within or outside the normal range, resulting in the actual decision if a hematologic OD episode is present or absent due to an off-limit condition.

### Data Models and Querying

**Fig. 3:**
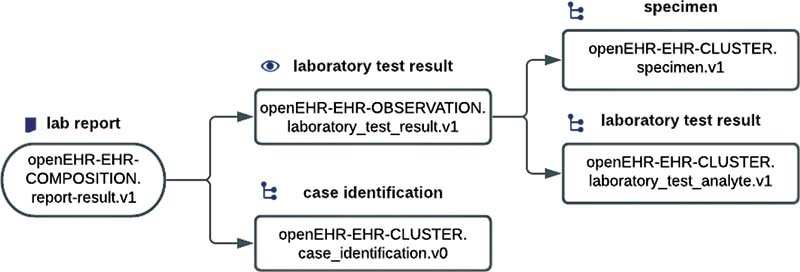
openEHR template for storing laboratory results (ID: ELISE Laboratory Report).

For this work, already existing archetypes could be reused to model three templates: (1) laboratory report, (2) diagnosis report, and (3) procedure report. The “laboratory report” ( Fig. 3 ) contains data related to laboratory findings. The “diagnosis report” stores the diagnoses of a patient, and the “procedure report” holds data about the performed procedures.

Since a “laboratory report” can store many test results, each instance of the cluster archetype “laboratory test result” stores, in addition to the laboratory value and its corresponding specimen collection timestamp, the terminology ID “LOINC” and its actual LOINC codes (platelets: “777–3,” INR: “6301–6”) in the corresponding data element of type DV_CODED_TEXT. The enrichment of LOINC terminology bindings ensures standardized data retrieval across different institutions, preconditioned that the AQL query is restricted to the same LOINC code (e.g., /name/defining_code/code_string = “777–3”).

Based on the template structure, we defined AQL queries to load data into the CDSS. In the first step, these queries were assigned to a variable in the CDSS, storing the query in a string format. Second, a REST API processed this string into a result set. Third, a loop iterated through each line of the result set and instantiates objects for each value of the patient ( *Patient State* ). Further, these facts could be inserted into Drools streams where rules enriched the attributes of the objects before transferring the generated knowledge into a target database.

### Implemented Knowledge Base, Reasoning Mechanisms, and User Interface

IPSCC diagnostic criteria serve as the foundation of the implemented rules (explicit knowledge). 3 However, some institution-specific adaptions to these internationally defined criteria were realized due to special characteristics of the patient collective of MHH. The first criterion was modified by using the first value after patient admission (here: baseline) as a comparator instead of the highest value recorded over the past 3 days to prevent adulterations associated with platelet transfusions or bleeding complications due to surgery. To decide whether a patient meets the baseline criterion, our domain experts identified diagnoses ( Supplementary Appendix B , available in the online version) to recognize if a patient suffers from a chronic hematological/oncological illness associated with thrombocytopenia. If the patient was diagnosed with at least one of the diagnoses, hematologic OD would be present if either the platelet count would be below a threshold of 80,000/mm ^3^ or if there would be a decline of 50% in platelet count compared with the first value recorded. Therefore, the latter criterion only applies to patients where at least one of these diagnoses was documented to catch the patients that might have platelet counts above 80,000/mm ^3^ , however, their count in platelets significantly dropped due to a possible hematologic OD.

Primary diseases associated with an increase in INR ( Supplementary Appendix B , available in the online version) have been identified, e.g., hereditary deficiencies. Analogous to the platelet count criterion, if the patient's diagnoses match with one of the ICD-10-GM codes (German Modification of the International Classification of Diseases) 39 listed in Supplementary Appendix B (available in the online version), an increased INR will not be taken into account for the application of further rules.

Additionally, our domain experts decided to distinguish between patients who need extracorporeal membrane oxygenation (ECMO) and patients who do not need ECMO, since the CDSS and the physician in charge should incorporate that information when diagnosing thrombocytopenia. To circumvent false positives associated with ECMO (e.g., due to the administration of heparin or after pains of ECMO), a time span of 2 hours pre-ECMO and 12 hours post-ECMO has been added to the duration of ECMO.

**Fig. 4:**
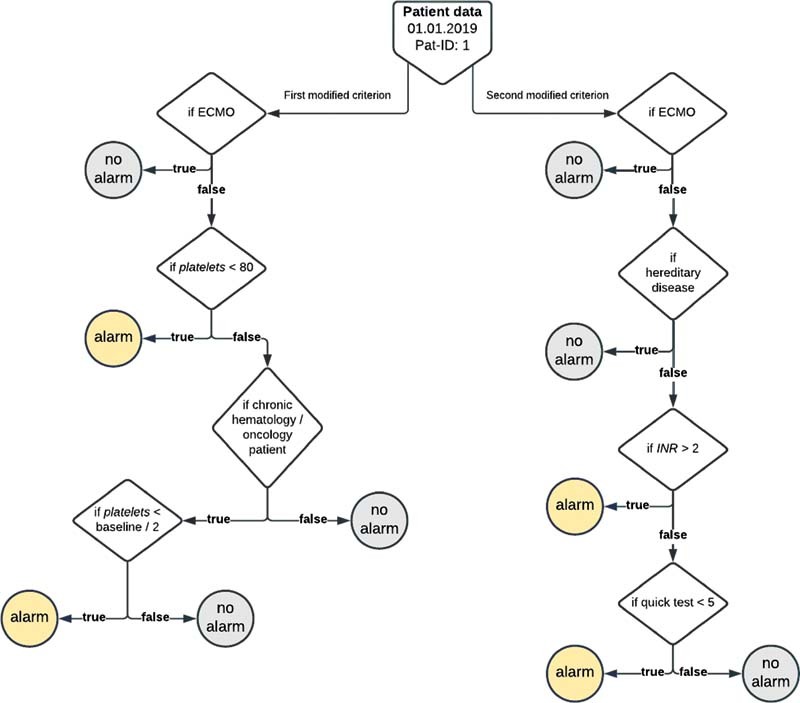
Reasoning process based on explicit and tacit knowledge assets.

Fig. 4 shows the reasoning process for the hematologic OD including all adoptions. The left branch indicates under which circumstances an alarm is elicited, caused by a lack in platelet count. The right branch encompasses the conditions leading to an alarm caused by an offset limit violation of INR. If the alarms occur simultaneously, they are being concatenated to one hematologic OD caused by both abnormal platelets and INR values.

**Fig. 5:**
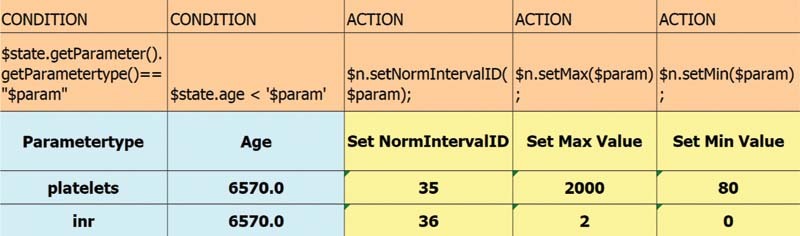
Extract of decision table for assigning the correct normal range to the patient's parameter value.

Drools decision tables are suitable for equally structured rules, in which each row represents a separate rule, whereas drools rule files encompass more specific rules. Each inference is associated with a set of rules which fire simultaneously. The rule engine ensures that another set of rules of the next inference can only fire until the previous inference step has been completed. First, a decision table makes sure that the incoming value is assigned to the corresponding normal range (parameter norm model). If every condition of a rule is met, the actions are being triggered. In this particular ruleset, the attributes maximum and minimum of the concept norm obtain a value, as shown in Fig. 5 . Note that the diagnostic criteria only suggest a minimum value for platelet count and a maximum value for INR. The respective other boundary value is based on clinically reasonable values provided by domain experts.

Subsequently, rules of three decision tables fire simultaneously using the knowledge generated by the *specify* -inference (alarm model). Depending on the parameter type and the presence of a limit value violation, at least one condition among all the rules of all three tables (distinguished by their alarm category) is met. In case the value exceeds the maximum (alarm category = “too high”) or falls below the minimum (alarm category = “too low”), this results in a so-called *alarm event* . Unlike these scenarios, the value can also be within the normal range (alarm category = “normal,” no alarm event is fired).

Based on the output of the decision tables, this newly generated knowledge can be used for further rules implemented via rule files. The ongoing inference mechanism is based on the step where *alarm events* of the same parameter type and alarm category merge if the events occur simultaneously. The resulting united *alarm event* has the starting point of the first *alarm event* and the ending point of the last overlapping *alarm event* .

**Fig. 6:**
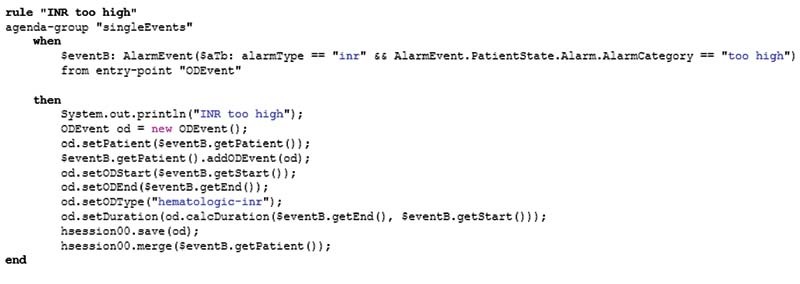
Example for eliciting an alarm due to increased INR implemented with Drools rule. INR, international normalized ratio.

Fig. 6 gives an example of the Drools syntax of a rule in which an INR alarm (when-condition) results in an OD event being of the type “hematologic-inr” (then-condition).

**Fig. 7:**
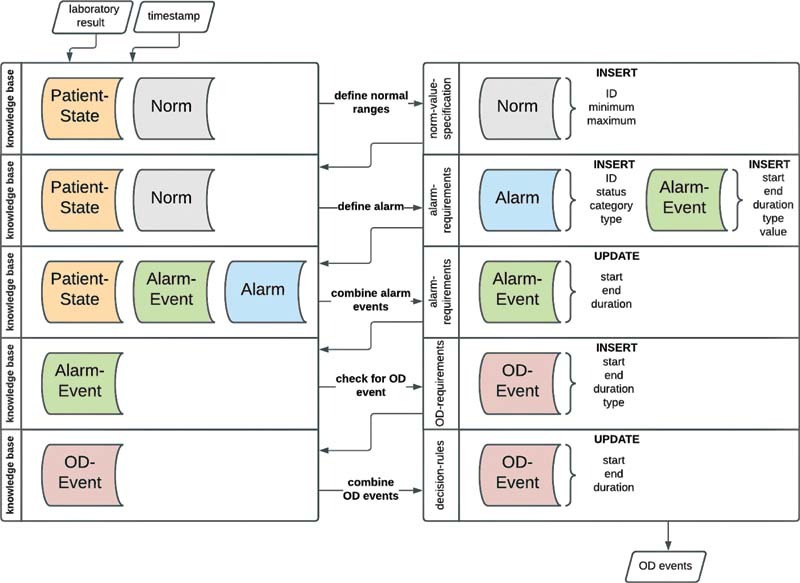
Iterative knowledge base alteration during reasoning.

To visualize the process of constantly changing the knowledge base throughout the reasoning process, Fig. 7 demonstrates which attributes of the concepts within the knowledge base are being enriched (right hand side) and how the knowledge base alters during application runtime (left hand side).

**Fig. 8:**
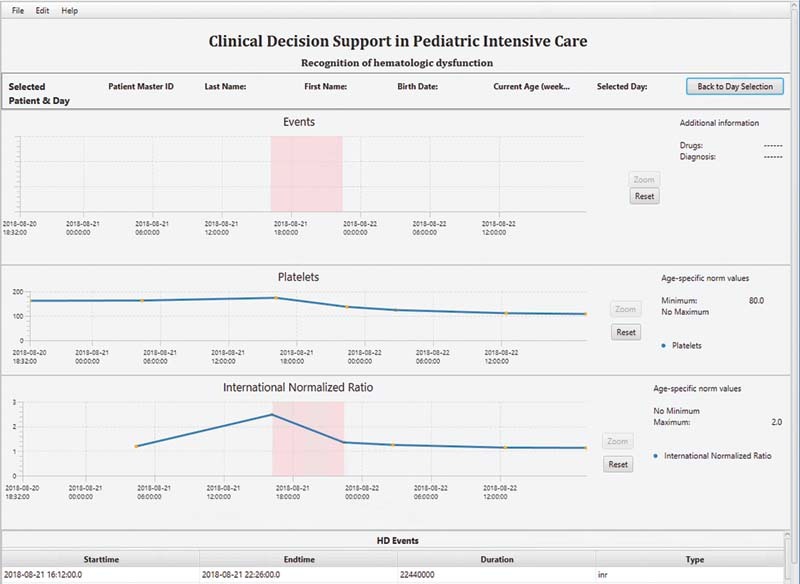
Graphical user interface for the recognition of hematologic dysfunction.

The final decision of the CDSS is delineated in a clear and precise manner so that medical staff can retrace and comprehend the CDSS decision (explanation facility). Charts visualize alarms by illustrating the time course of the parameter values as well as emphasizing the presence of an OD episode in red color. Next to each chart, the normative values for the patient are visible to explain CDSS decisions. A table at the bottom summarizes relevant information of the alarm such as duration, parameter type, start and end time point. To align with the existing CDSS design, presented by Wulff et al, 20 the graphical user interface has the same appearance ( Fig. 8 ).

### Estimation of Diagnostic Accuracy

**Fig. 9:**
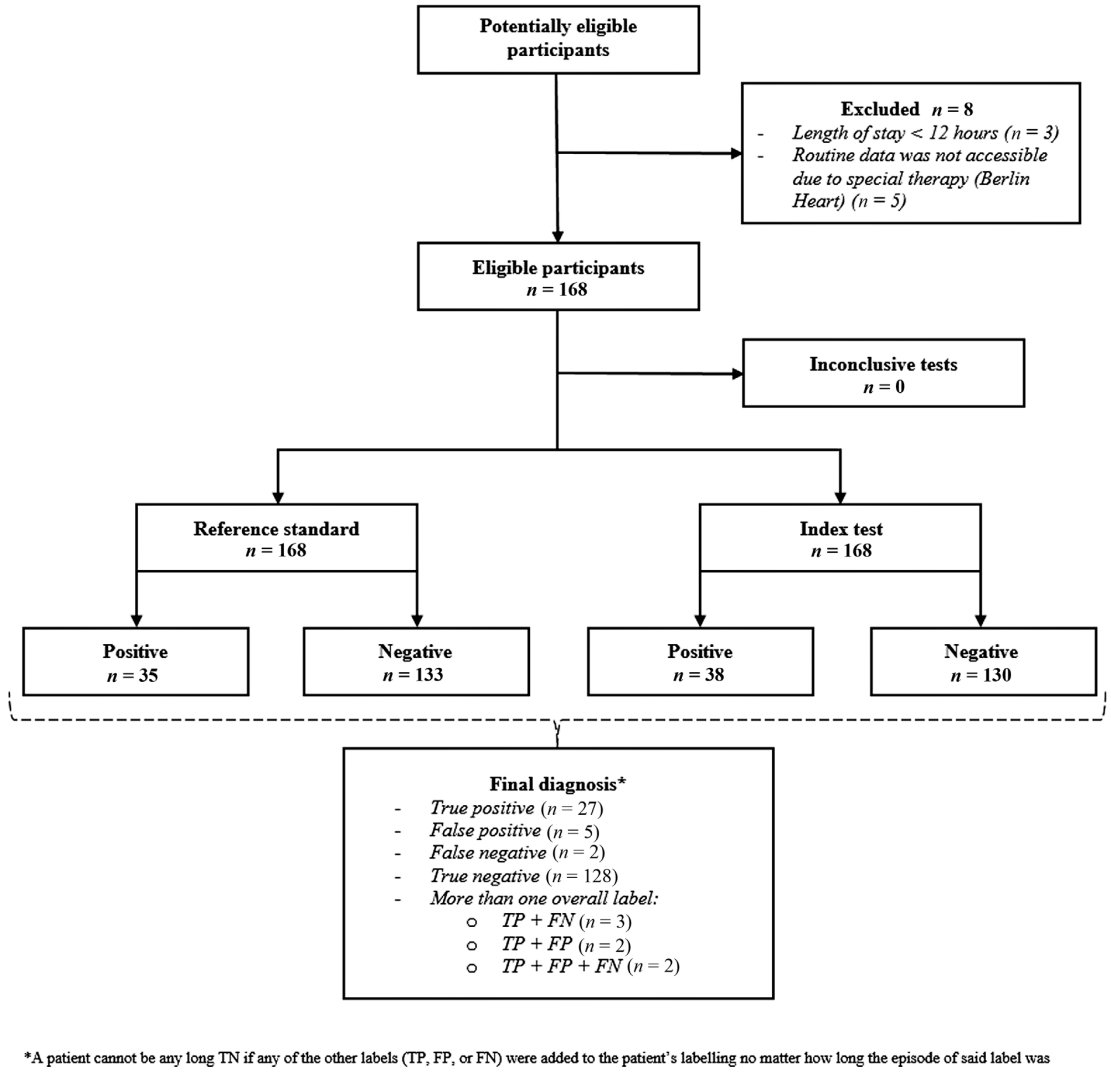
Flow diagram for recruited patients including their overall hematologic OD label (for details on the overall label see Supplementary Material S1 (available in the online version).

The data for PoC included 168 patients (0–18 years) with 1.998 admission days and 337 blocks. Overall, 67 episodes of hematologic OD in 35 patients (10 patients with ≥two episodes) were recorded by the clinicians. The rule-based CDSS detected the onset of the hematologic dysfunction with a sensitivity of 0.821 (95% CI: 0.708–0.904) and a specificity of 0.970 (95% CI:0.942–0.987) ( Table 1 ). The research did not cause any adverse events for patients, because the index test was applied retrospectively ( Fig. 9 ).

## Discussion

### Strengths and Limitations

We have shown that our interoperable CDSS concept, first introduced for SIRS detection in children, is transferrable to other relevant clinical use cases. Addressing some false positives, one limitation of the CDSS is its inability to recognize pre-analytical errors, e.g., improper sample collection, or post-analytical errors such as mismatched laboratory specimens. However, this limitation could be addressed in a rule that compares each abnormal value with the previous and following value within a pre-defined timeframe. The timeframe should be selected in a way that would indicate that the clinician suspects an analytical error, hence ordering a new laboratory test immediately. Therefore, if the following value is not pathological and significantly differs from the current, pathological value, it is likely to be an erroneous value that does not fit the constitution of the patient. Other false positives are due to other procedures, especially if the patient has lost high volumes of blood during surgery, resulting in low platelet counts. The false negatives are related to the ECMO rules. In specific cases, for example, when the patient takes a relatively long time to achieve normal platelet levels after ECMO or achieves it only with repeated administration of platelets, clinicians suspect a hematologic dysfunction, deviating from the criteria. Another reason why sensitivity on the patient level was not as good as expected could be the quality of the primary source data. Overall, several strategies will be developed to optimize the CDSS before further steps are taken.

We are aware that our sample size of 168 patients is relatively small, which is related to the fact that, first, pediatric cohorts are generally smaller and, second, no reference standard with episodes of SIRS/sepsis and associated OD is available. The reason why we have not evaluated our approach on other wards is that a continuous, digitalized documentation of vital signs, laboratory values, and other values is a rarity in German wards. Without these data in other settings, it is simply not possible to use such a computerized clinical decision support approach for SIRS/sepsis and associated ODs. Consequently, we were not able to evaluate the transferability of our system to other wards. Nevertheless, we will integrate measured values from the intermediate care units shortly. This will be the first step to test our system also for other patient cohorts with other characteristics.

Rather than simply detecting abnormal laboratory values similar to what laboratory reporting systems are capable of, our goal was to detect SIRS/sepsis-associated hematologic dysfunction according to the IPSCC criteria, since such dependencies are not implemented in our laboratory information system. Additionally, some diagnostic criteria were modified due to specifics in MHH diagnostics and data capture. Consequently, these criteria differ from the consensus criteria defined by multiple experts from the IPSCC. To address this, the CDSS could be enhanced by user input fields to be able to choose from different available criteria to adapt to site-specific conditions. Further adaptions before the CDSS will be implemented in the PDMS, include, for example, integrating warnings when ECMO is present in the patient. In this case, the system should warn the pediatrician about abnormal values to prevent any fatal missing conditions of the patient on ECMO but will not label it as a dysfunction, unless the pediatrician determines otherwise. In general, the CDSS would have to be enriched with further knowledge to create alerts with clinical relevance as just described, but which do not lead to a diagnosis.

Relying on open standards (e.g., openEHR) is a clear advantage since it is vendor-neutral and openly specified. Another strength of the CDSS is its ability to produce high-quality labeled datasets useful to train machine-learning models. Once the CDSS can detect dysfunction of other organ systems, it provides data scientists with an efficient and fast-processing tool for data annotation concerning the multi-class problem of MODS. Since the prototypical approach had proven beneficial, it will serve as a role model for future implementations of dysfunction or failure of other organ systems along the pathway of SIRS/sepsis. These future implementations also include implementation into routine work, once our approach was proven beneficial in a multicenter study. For this, we are working closely together with a certified manufacturer to implement our results to comply with regulations of the European Medical Device Regulation (MDR). Thus, this work is intended to share early results with interested scientists. However, it is worthwhile to mention that our algorithm was applied to real clinical routine data.

As explained, our work is primarily about detecting SIRS/sepsis-associated hematologic dysfunctions. To differentiate between SIRS/sepsis-associated pathologic states and underlying diseases also resulting in abnormal IPSCC parameters, we implemented ICD-10-based measures. Indeed, since we decided to focus SIRS/sepsis progression, our approach is limited because detection of OD from other causes is not integrated.

### Future Directions

We were able to present promising findings for data-driven hematologic OD detection by reusing an interoperable CDSS approach. In one of the next steps, we also aim at evaluating our approach in other, more general pediatric wards by making use of our labeling mechanism. In this context, we focus on labeling a large dataset of pediatric intensive care patients (approximately 5,000 encounters from 2015 to 2021) to use it as training and test data for data-driven approaches, which will also allow evaluating algorithms on a broader basis. For more information, we would like to refer to the publication server of our dataset. 40 We are currently in the process of further enriching the knowledge base with rules regarding more complex OD such as hepatic, renal, cardiovascular, and respiratory OD, dealing with more parameters and age-specific differences. In parallel, the PDMS manufacturer of MHH is implementing the rules that have already been evaluated in their certified medical device. Thereafter, we can make a definite statement on whether the diagnostic accuracy yields similar results to what we yielded with our CDSS in one of the first steps of CDSS implementation.

In addition, we aim at publishing a completely open accessible demonstrator, which will be published in a couple of weeks, so that the source code can be optimized by each interested researcher. 41 The next stage of our research will be the implementation of combination rules to detect MODS. Additional work on acquiring knowledge for organ failure would help us to implement rules for the detection of organ failure in critically ill children. Research into developing machine-learning algorithms using the labeled dataset by our CDSS is also underway, as questions about a potential refinement of the diagnostic criteria can be raised. Moreover, a usability study is planned and will be conducted in the upcoming months. We will assess the usability of our system by using quantitative and qualitative assessments, amongst others, resulting in a final SUS score. 42

## Conclusion

The authors have shown that our interoperable CDSS concept is transferrable to other clinical use cases. Using a stepwise approach, we augmented our CDSS with the capability for the detection of pediatric hematologic OD. The results of our evaluation show that the CDSS will correctly diagnose 82% of the patients who have a hematologic OD, but the CDSS will also issue a diagnosis for 3% of the patients who do not have a hematologic OD. The practicability concerning more complex OD will be addressed in future work.

## Clinical Relevance Statement

Health care professionals will benefit from CDSS if these systems consolidate information from different source systems, analyze a large amount of heterogeneous data, present the most important pieces of information, and enable accurate, fast, and informed decision-making even in time-critical and high-risk situations. The ability of CDSS to detect OD can have a direct impact on the workload of pediatric intensive care physicians, including lower stress levels and error prevention, as well as it can support inexperienced physicians and close knowledge gaps. With CDSS for early detection of OD, the physician will be able to quickly assess the patient's condition and decide on further treatments that would better match the patient's risk status.

## Multiple Choice Questions

1. Which of the following is a well-known bottleneck of CDSS adoption?
  - The design of new CDSS as standalone systems.
  - The use of semantic and syntactic interoperability standards.
  - The design of new CDSS with interfaces to EHRs or further primary source systems in the information system landscape of the institution.
  - The reuse of existing routine data captured in primary source documentation or monitoring systems.
    **Correct Answer:** The correct answer is option a. Stand-alone CDSS lacks interoperability and can disrupt the physician's workflow when not connected to the systems used in daily routine.
2. Which of the following aspects is decisive in the application of the openEHR approach for semantic modeling and structured representation of clinical concepts?
  - A deep technical understanding of the underlying database structure.
  - The reuse of existing archetypes that already have been published in freely accessible repositories.
  - Advanced knowledge of a programming language (e.g., Java, C + +).
  - The use of a specific vendor-dependent commercial archetype designer.
    **Correct Answer:** The correct answer is option b. Many clinical concepts are already available in international archetype repositories (e.g., Clinical Knowledge Manager, CKM, www.openehr.org/ckm ). These archetypes are used in other openEHR-projects and have been developed in collaboration with various international experts. Reusing such archetypes not only preserves international interoperability but also saves resources. In addition, modeling is not limited to a specific commercial tool, as various products, including open source solutions, exist. Finally, openEHR realizes *two-level modeling* so that no deep understanding of technical problems such as the underlying database structure or advanced knowledge in a programming language is required.

## Footnotes

a
*https://medisite.de/mlife/.Protection of Human and Animal Subjects*
