# Supplementary material for: Tracing the Progression of Sepsis in Critically Ill Children: Clinical Decision Support for Detection of Hematologic Dysfunction
Source: Appl Clin Inform. 2022 Oct 26;13(5):1002–14. doi: 10.1055/a-1950-9637 (PMC9605821; doi:10.1055/a-1950-9637)
Supplement: Supplementary file 1 — Supplementary Material [file 10-1055-a-1950-9637-s202203ra0079.pdf]

Supplementary Appendix A Elise Study Group Members

Louisa Bode[1]; Marcel Mast[1]; Antje Wulff[1,2]; Michael Marschollek[1]; Sven Schamer[3]; Henning Rathert[3]; Thomas Jack[3]; Philipp Beerbaum[3]; Nicole Rübsamen[4]; Julia Böhnke[4]; André Karch[4]; Pronaya Prosun Das[5]; Lena Wiese[5]; Christian Grosz-weski-Anders[6]; Andreas Haller[6]; Torsten Frank[6]

[1]Peter L. Reichertz Institute for Medical Informatics of TU Braunschweig and Hannover Medical School, Hannover, Germany

[2]Big Data in Medicine, Department of Health Services Research, School of Medicine and Health Sciences, Carl von Ossietzky University Oldenburg, Oldenburg, Germany

[3]Department of Pediatric Cardiology and Intensive Care Medicine, Hannover Medical School, Hannover, Germany

[4]Institute of Epidemiology and Social Medicine, University of Muenster, Muenster, Germany

[5]Research Group Bioinformatics, Fraunhofer Institute for Toxicology and Experimental Medicine, Hannover, Germany

[6]Medisite GmbH, Hannover, Germany

Supplementary Appendix B ICD-10-GM Codes for chronically hematology/oncology patients

Parameter/ Criterion	ICD code	German description [English description]
Platelet count	D59.3	Hämolytisch-urämisches Syndrom [engl. Hemolytic-uraemic syndrome]
	D69.3	Idiopathische thrombozytopenische Purpura [engl. Idiopathic thrombocytopenic purpura]
	D69.4	Sonstige primäre Thrombozytopenie [engl. Other primary thrombocytopenia]
	D69.40	Sonstige primäre Thrombozytopenie, als transfusionsrefraktär bezeichnet[engl. Other primary thrombocytopenia designated as transfusion refractory]
	D69.41	Sonstige primäre Thrombozytopenie, nicht als transfusionsrefraktär bezeichnet [engl. Other primary thrombocytopenia not designated as transfusion refractory]
	D69.5	Sekundäre Thrombozytopenie [engl. Secondary thrombocytopenia]
	D69.52	Heparin-induzierte Thrombozytopenie Typ I [engl. Heparin-induced thrombocytopenia type I]
	D69.53	Heparin-induzierte Thrombozytopenie Typ II [engl. Heparin-induced thrombocytopenia type II]
	D69.57	Sonstige sekundäre Thrombozytopenien, als transfusionsrefraktär bezeichnet [engl. Other secondary thrombocytopenias designated as transfusion refractory]
	D69.6	Thrombozytopenie, nicht näher bezeichnet [engl. Thrombocytopenia, unspecified]
	D69.60	Thrombozytopenie, nicht näher bezeichnet, als transfusionsrefraktär bezeichnet [engl. Thrombocytopenia, unspecified, designated as transfusion refractory]
	D73.1	Hypersplenismus [engl. Hypersplenism]
	D73.2	Chronisch-kongestive Splenomegalie [engl. Chronic congestive splenomegaly]
	D82.0	Wiskott-Aldrich-Syndrom [engl. Wiskott-Aldrich syndrome]
	M31.1	Thrombotische Mikroangiopathie [engl. Thrombotic microangiopathy]
	Q87.2	Angeborene Fehlbildungssyndrome mit vorwiegender Beteiligung der Extremitäten [engl. Congenital malformation syndromes predominantly involving limbs]
INR	D67	Hereditärer Faktor-IX-Mangel [engl. Hereditary factor IX deficiency]
	D68.2	Hereditärer Mangel an sonstigen Gerinnungsfaktoren [engl. Hereditary deficiency of other clotting factors]
	D68.20	Hereditärer Faktor-I-Mangel [engl. Hereditary factor I deficiency]
	D68.21	Hereditärer Faktor-II-Mangel [engl. Hereditary factor II deficiency]
	D68.22	Hereditärer Faktor-V-Mangel [engl. Hereditary factor V deficiency]
	D68.23	Hereditärer Faktor-VII-Mangel [engl. Hereditary factor VII deficiency]

Abbreviation: INR, international normalized ratio.

 © 2022. The Author(s).

Supplementary Material S1

Assessment of clinical decision support systems (CDSS) within the Elise project

Definitions

True positive (TP)	At a given time point during the PICU stay, both the reference standard and the CDSS detect the occurrence of the target disease.
False positive (FP)	At a given time point during the PICU stay, the CDSS detects the occurrence of the target disease, but the reference standard does not.
False negative (FN)	At a given time point during the PICU stay, the reference standard detects the occurrence of the target disease, but the CDSS does not.
True negative (TN)	At a given time point during the PICU stay, both the reference standard and the CDSS do not detect the occurrence of the target disease.
Sensitivity	$\Sigma TP / (\Sigma TP + \Sigma FN)$, i.e., probability of a positive index test (CDSS) given that the reference standard detects the occurrence of the target disease.
Specificity	$\Sigma TN / (\Sigma TN + \Sigma FP)$, i.e., probability of a negative index test (CDSS) given that the reference standard does not detect the occurrence of the target disease.

Labeling

Per patient and PICU stay, each block (i.e., subunit of the stay generated dependent on a change in the diagnostic status [OD present vs. no OD] of either the CDSS, the reference standard, or both simultaneously) was labeled as either true positive (TP), false positive (FP), false negative (FN), or true negative (TN) (see ►**Supplementary Fig. S1**).

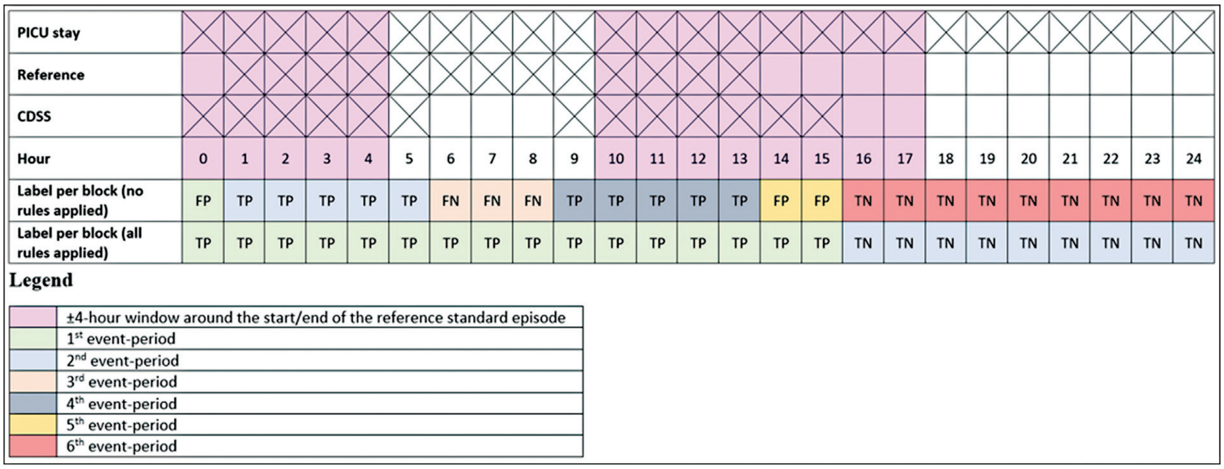

Supplementary Fig. S1. Example for the labels per patient.

In addition, four rules were applied for the labeling:

- *±4-hour window:* The CDSS diagnostic episode start or end is TP if the CDSS diagnostic episode start or end happened within the ±4-hour window (which corresponds to a shift of 8 hours) around the start or end of the reference standard diagnostic episode. Hence, FN or FP within this time window was converted to TP.
- *Merging:* Diagnostic episodes with less than 24 hours in-between the end of one diagnostic episode and the start of a new diagnostic episode were merged into one (this is done before labeling). Gaps in the CDSS episode (while the diagnostic episode of the reference standard is ongoing) are possible if more than 24 hours remain between the CDSS diagnostic episode end and the start of a new CDSS diagnostic episode.
- *Death:* The death of a patient is equal to end of the last episode of that particular patient. Furthermore, it is equal to the discharge of the patient.
- *PICU stay:* Diagnostic episodes before or after the pediatric intensive care unit (PICU) stay were excluded from the diagnostic test accuracy estimation.

 © 2022. The Author(s).

Overall Labeling (As Shown in Patients' Flow Chart)

The overall patient label of a PICU stay is derived from the application of the following rules:

- *TN*: The overall label of a PICU stay of a patient is *TN* if the patient did not experience a diagnostic episode (neither a CDSS episode nor a reference standard episode) during this specific PICU stay, i.e., all hours of this PICU stay are labeled as *TN*.
- *TP*: The overall label of a PICU stay of a patient is *TP* if the patient has at least one block that is labeled as *TP* during this specific PICU stay. Furthermore, for this patient, no block of this specific PICU stay is labeled as *FN* or *FP*.
- *FP*: The overall label of a PICU stay of a patient is *FP* if the patient has at least one block that is labeled as *FP* during this specific PICU stay. Furthermore, for this patient, no block of this specific PICU stay is labeled as *FN* or *TP*.
- *FN*: The overall label of a PICU stay of a patient is *FN* if the patient has at least one block that is labeled as *FN* during this specific PICU stay. Furthermore, for this patient no block of this specific PICU stay is labeled as *TP* or *FP*.
- *TP* + *FN*: The overall label of a PICU stay of a patient is "*TP* + *FN*" if the patient has at least one block that is labeled as *TP* and at least one block that is labeled as *FN* during this specific PICU stay. Furthermore, for this patient, no block of this specific PICU stay is labeled as *FP*.
- *TP* + *FP*: The overall label of a PICU stay of a patient is "*TP* + *FP*" if the patient has at least one block that is labeled as *TP* and at least one block that is labeled as *FP* during this specific PICU stay. Furthermore, for this patient no block of this specific PICU stay is labeled as *FN*.
- *TP* + *FP* + *FN*: The overall label of a PICU stay of a patient is "*TP* + *FP* +*FN*" if the patient has at least one block that is labeled as *TP* and at least one block that is labeled as *FP* and at least one block that is labeled as *FN* during this specific PICU stay.

Diagnostic Test Accuracy (DTA) Estimation

Based on the block labels, sensitivity and specificity with their 95% confidence intervals were estimated using the approach by Brunner and Zapf, Lange, and Rooney.[1–3] This approach accounts for the longitudinal data format by weighting each event. However, the corresponding R package currently cannot estimate the lower and upper bound of the 95% confidence interval (CI) if there is a disbalance of cluster groups (i.e., "at least one group in a dependent block of units contains less than two units"). Also, the package was able to estimate the weighted point estimation for the sensitivity and specificity, the 95% CI needed to be manually computed, i.e., they are not weighted.

DTA of Hematologic Dysfunction

This DTA estimation used only the rules as stated in *LABELING*; hence, this estimation assessed both, the correctness of the episode starts and episode ends. Here, the rule-based CDSS diagnostic performance estimated the DTA of the hematologic dysfunction with a sensitivity of 0.821 (95% CI: 0.708–0.904) and a specificity of 0.970 (95% CI: 0.942–0.987). The sensitivity of the CDSS was mainly influenced by either too short (causing FN events) or too long (causing FP events) diagnostic CDSS episodes compared with the reference standard, or an ongoing reference standard diagnostic episode while the CDSS diagnostic episode labeled at least one period as disease-free ("gap") during the ongoing reference standard episode.

Episode Correctness Assessment

This part assessed the correctness of the CDSS episodes compared with the reference standard episodes by removing all rules except the merging of episodes smaller than 24 hours from the analysis.

The episode start or episode end was correct if the CDSS episode and the reference standard episode started or ended simultaneously (i.e., date and time are identical). ►**Supplementary Fig. S2** summarizes the frequency of correct and incorrect episode starts and episode ends as measured with the hematologic dysfunction. This summary showed that the CDSS episode start was mostly correct, while the CDSS episode end was less often coincided with the end of the reference standard.

 © 2022. The Author(s).

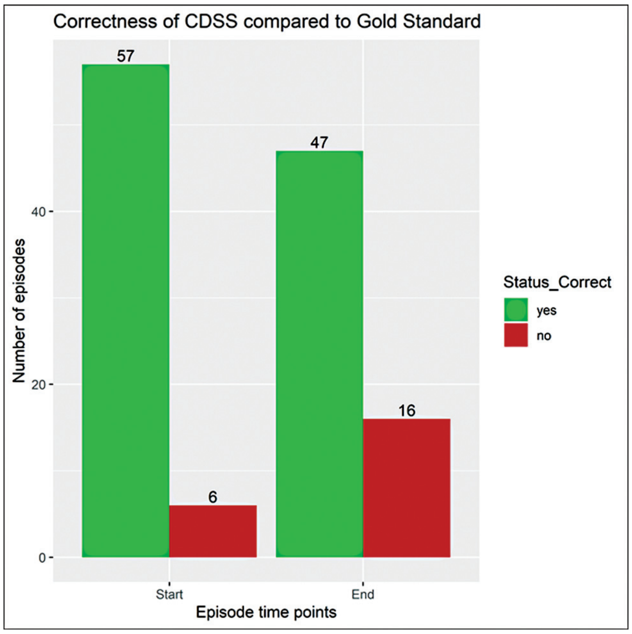

Supplementary Fig. S2 Correctness of episode start and episode end.

Further, research on the incorrectness of the CDSS episode start and episode ends was conducted by calculating the difference in hours between episode starts and episode ends between the CDSS and the reference standard. These differences were then grouped (i.e., "0–4," "4–8," "8–12," "12–16," "16–20," "20–24," "24–48," "48–62," "62–86," and "86 + " hours) according to their timely difference and which of the raters first started or ended the episode. ►Supplementary Figs. S3 and S4 summarize the difference between the CDSS and the reference standard for the episode start and episode end, respectively. Notice that episode differences of $\geq$24 hours were associated with gaps in the CDSS compared with the reference standard episode.

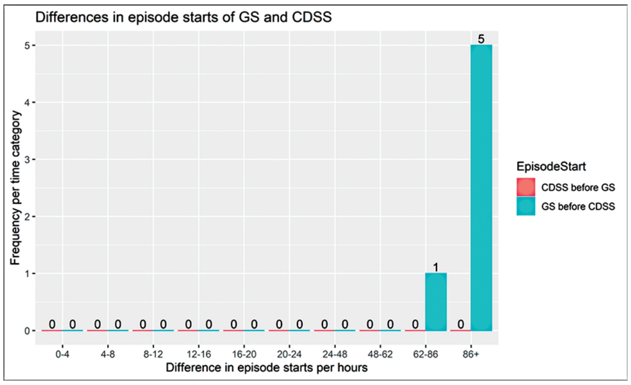

Supplementary Fig. S3 Differences in episode starts.

 © 2022. The Author(s).

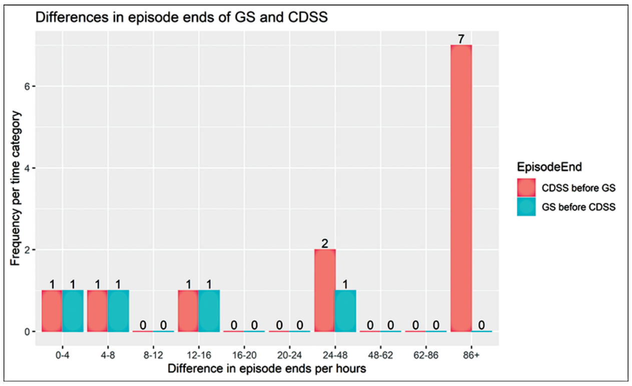

Supplementary Fig. S4 Differences in episode ends.

CDSS gaps occur when the CDSS produced multiple (at least two) diagnostic episode onsets during the period where the reference standard episode was still ongoing. ►Supplementary Fig. S5 summarizes the occurrence of CDSS gaps and ►Supplementary Fig. S6 the length of gaps. Note that the length of CDSS gaps cannot be less than 24 hours due to the merging rule. All other gap lengths were categorized according to their length in hours (i.e., "24–48," "48–62," "62–86," and "86 + " hours). These CDSS gaps were mostly associated with an improved health status of the patient (e.g., due to treatment or spontaneous recovery of the patient).

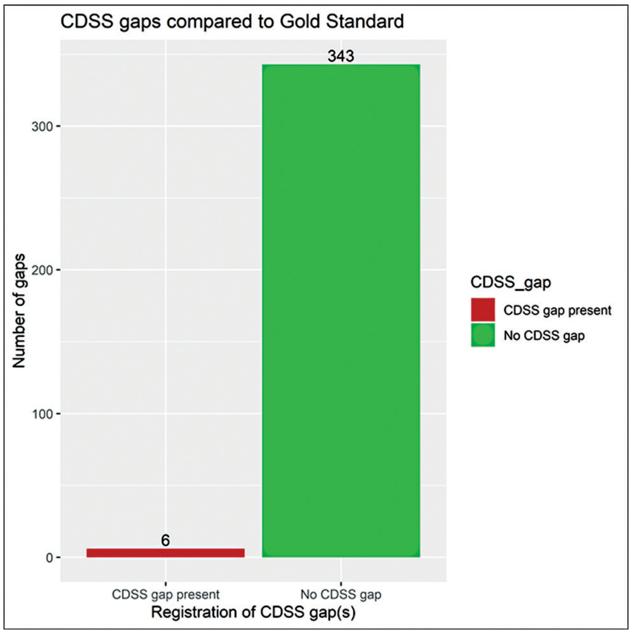

Supplementary Fig. S5 Occurrence of CDSS gaps compared with reference standard, CDSS, clinical decision support systems.

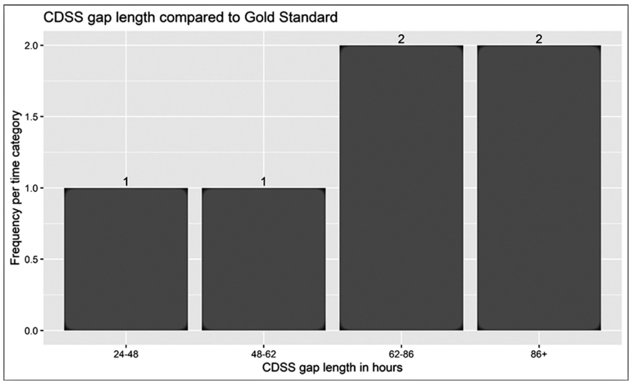

Supplementary Fig. S6 Length of CDSS gap in hours. CDSS, clinical decision support system.

References

1 Brunner E, Zapf A. Nonparametric ROC analysis for diagnostic trials. Methods Appl Stat Clin Trials 2014;2:483–495
2 Lange K. Nichtparametrische Analyse diagnostischer Gütemaße bei Clusterdaten. Georg-August University Göttingen; 2011
3 Rooney D. Covariate Adjusted Nonparametric Estimation of Sensitivity and Specificity. Ruprecht-Karls University Heidelberg; 2017

 © 2022. The Author(s).
